# Determining the cut-off value for the Minimal Documentation System (MIDOS2) screening tool to initiate specialized palliative care based on patient’s subjective need for palliative support and symptom burden in inpatients with advanced cancer

**DOI:** 10.1007/s00432-024-05897-x

**Published:** 2024-07-24

**Authors:** Anna Heinzelmann, Mitra Tewes, Sandy Müller, Ulrich Sure, Ken Herrmann, Dirk Schadendorf, Eva Warnecke, Raya Rausch, Eva-Maria Skoda, Maria Rosa Salvador Comino

**Affiliations:** 1 Department of Palliative Medicine, University Hospital Essen (AöR), 45147 Essen, Germany; 2 Department of Neurosurgery, University Hospital Essen (AöR), 45147 Essen, Germany; 3 Department of Nuclearmedicine, University Hospital Essen (AöR), 45147 Essen, Germany; 4 Department of Dermatology, University Hospital Essen (AöR), 45147 Essen, Germany; 5 West German Cancer Center, University Hospital Essen (AöR), 45147 Essen, Germany; 6 Department for Psychosomatic Medicine and Psychotherapy LVR-University Hospital Essen, University of Duisburg-Essen, Essen, Germany; 7 Center for Translational Neuro-and Behavioral Sciences (C-TNBS), University of Duisburg-Essen, Essen, Germany

**Keywords:** Palliative care, Multidimensional assessment, ePROM, Screening, Cancer patients, MIDOS2

## Abstract

**Purpose:**

The Minimal Documentation System (MIDOS2) is recommended as a systematic screening tool for assessing symptom burden and patient needs in advanced cancer patients. Given the absence of an optimal weighting of individual symptoms and a corresponding cut-off value, this study aims to determine a threshold based on inpatient’s subjective need for palliative support. Additionally, we investigate the correlation between symptom burden and subjective need for palliative support collected through a patient-reported outcome measure (PROM) with survival duration of less or more than one year.

**Methods:**

Inpatients diagnosed with advanced solid cancer completed an electronic PROM, which included the MIDOS2 questionnaire among other tools. Differences in symptom burden were analysed between patients expressing subjective need for palliative support and those with survival of less or more than one year using ANOVA, Mann–Whitney-U Test, logistic regression, Pearson and Spearman correlation tests. Cut-off analyses were performed using a ROC curve. Youden-Index, sensitivity, and specificity measures were used as well.

**Results:**

Between April 2020 and March 2021, 265 inpatients were included in the study. Using a ROC curve, the MIDOS2 analysis resulted in an Area under the curve (AUC) of 0.732, a corresponding cut-off value of eight points, a sensitivity of 76.36% and a specificity of 62.98% in assessing the subjective need for palliative support. The MIDOS2, with double weighting of the significant symptoms, showed a cut-off value of 14 points, achieving a sensitivity of 78.18% and a specificity of 72.38%. A total of 55 patients (20.8%) expressed a need for support from the palliative care team. This need was independent of the oncological tumour entity and increased among patients with a survival of less than one year. These patients reported significantly poorer physical (p < 0.001) or mental (p < 0.001) condition. Additionally, they reported higher intensities of pain (p = 0.002), depressive symptoms (p < 0.001), weakness (p < 0.001), anxiety (p < 0.001), and tiredness (p < 0.001).

**Conclusion:**

Using the established MIDOS2 cut-off value with an adjusted double weighting in our study, a large proportion of inpatients may be accurately referred to SPC based on their subjective need for palliative support. Additionally, subjective reports of poor general, mental, and physical condition, as well as pain, depressive symptoms, weakness, anxiety, and tiredness, increase the subjective need for palliative support, particularly in patients with a survival prognosis of less than one year.

**Supplementary Information:**

The online version contains supplementary material available at 10.1007/s00432-024-05897-x.

## Introduction

The benefits of early identification and engagement of specialized palliative care (SPC) during the treatment of advanced malignancies have been extensively documented and supported by numerous studies (Schlick and Bentrem 2019; Milazzo et al. 2020; Haun et al. 2017) especially for cancer patients with a prognostic survival of less than 12 months or experiencing a high symptom burden (Vanbutsele et al. 2018; Follmann 2020; Kaasa et al. 2018). However, the optimal timing for SPC referral among advanced cancer patients (ACP) remains unclear, with referrals predominantly occurring late in the disease trajectory. This results in underutilization of the full benefits offered by palliative care (PC) (Lundeby et al. 2022; Jordan et al. 2020).

ACP commonly suffer from a wide variety of physical symptoms, as well as social and psychological distress (Vogt et al. 2021; Newcomb et al. 2020). Therefore, both national and international guidelines advocate for the documentation of patients’ needs as an integral component of routine clinical practice (Kaasa et al. 2018; Tewes et al. 2021; Baratelli et al. 2019). The assessment of symptom burden in ACP is typically conducted by health care professionals; however, this external approach often fails to identify important symptoms early enough, leaving them unaddressed or inadequately treated (Basch et al.^2020^; McGuire et al. 2016; Warnecke et al. 2023).

In a PC model where patients’ needs serve as the main criterion for SPC referrals, patient-reported outcome measures (PROMs) are widely used and recommended. These measures can recognize symptoms earlier, identify their severity, and highlight patients’ needs (Dawson et al. 2010). Furthermore, PROMs have been shown to improve communication between patients and healthcare providers, while also aiding in symptom management (Eid et al. 2017; Etkind et al. 2015). According to the German Guideline for Palliative Care (Follmann 2020), the Minimal Documentation System (MIDOS2) and the Integrated Palliative care Outcome Scale (IPOS) are suggested as systematic screening tools for assessing symptom burden and patients’ needs within clinical practice (Murtagh et al. 2019).

Various studies have documented threshold values using the Edmonton Symptom Assessment Scale (ESAS) for symptoms of clinical relevance (Hui and Bruera 2017). A recent large-scale study by Braulke et al. demonstrated that using different MIDOS2 sum scores (comprising four, five, or six symptoms, often in combination with at least one or two symptoms self-rated as severe or indication of a severely reduced general condition), a large percentage of patients showed a positive screening result, indicative of unmet palliative needs (Braulke et al. 2023). However, the aforementioned PROMs do not provide diagnostic capabilities or specific cut-off values for SPC referral, which could facilitate the early identification of patients with psycho-oncological and PC needs, challenging potential SPC referrals (Braulke et al. 2023).

Given the nationwide use of MIDOS2 (Braulke et al. 2023) as a systematic screening tool for evaluating symptom burden and patient needs across Germany, our primary aim was to determine the optimal weighting of the MIDOS2, stablish its ideal cut-off value, and evaluate its effectiveness in predicting the subjective need for palliative support experienced by ACP at a major German Comprehensive Cancer Centre. The secondary aim of this study was to correlate the symptom burden of these patients by using an electronic version of PROM (ePROM), including their subjective need for palliative support and survival of less or more than one year.

## Methods

**Fig. 1:**
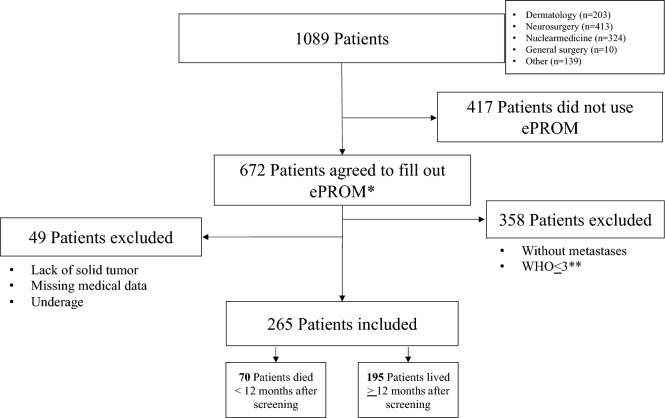
Results for the process of recruitment and enrolment of patients. *ePROM: electronic patient reported outcome measure. **WHO ≤ 3: tumor stage ≤ 3 according to the World Health Organization (WHO) Classification of Tumors

We conducted a retrospective, monocentric observational study including patients from a German Comprehensive Cancer Centre, based on our routine implementation of ePROM. We identified a total of 1,089 cancer patients treated across various specialized oncology wards, with the most prevalent being dermatology, neurosurgery, nuclear medicine, and general surgery. These patients were invited to use ePROM for the assessment of physical and psychological symptoms during the period from April 1st, 2020, to March 31st, 2021. Among them, 672 consented to participate. We excluded 407 patients (out of 672) for the following reasons: absence of a solid tumour (n = 29), missing relevant medical data (n = 13), age below 18 years (n = 7), and absence of metastatic disease or a tumor stage ≤ 3 according to the World Health Organization (WHO) Classification of Tumors (n = 358). Therefore, a total of 265 patients met the eligible criteria for inclusion in our study. Among these, 70 patients died within a year after the screening, while 195 patients survived more than one year after screening (Fig. 1).

### Study design and study population

We collected patient demographics and characteristics through a comprehensive review of medical records, from where gender, age, Eastern Cooperative Oncology Group (ECOG) (Conill et al. 1990), cancer diagnosis, histological type and stage were obtained. Patients had to meet the following inclusion criteria: age above 18 years, inpatient admission during the study, histologically confirmed cancer with metastases (UICC-stage IV), or a tumor stage > 3 according to the World Health Organization (WHO) Classification of Tumors and a willingness to complete the digital self-screening via ePROM. We obtained the date of death from the residents’ registration office for all patients. Patients were classified in two groups: the first group was defined based on the patient’s subjective need for palliative support. The second group was based on patient survival after screening: those with a survival of less than one year after screening and those with a survival of more than one year after screening. Please note, that the question in our PROM has been intentionally simplified to accommodate the fact that most patients are not well-versed in distinguishing between general and specialized palliative care. In our study, when a patient expressed the need for palliative support, they were visited by a member of the SPC team.

### ePROM (electronic patient reported outcome measure)

Our version of ePROM consists of a self-answered digital questionnaire known as ePOS (electronic psycho-oncological and palliative care screening), designed to assess both physical symptoms and psychological burden. The digital tablet-based questionnaire is distributed by nurses in both inpatient and outpatient settings. Our work focuses on the inpatient setting. After completion of the questionnaire, patients return the tablet to the nurse, and the provided information is transferred digitally to the hospital's internal electronic patient information system, thereby facilitating access for hospital health care workers and leaving the resulting intervention to the discretion of the treating team.

The ePROM comprise five validated, multidimensional assessment questionnaires for symptoms and needs: the Personal Health Questionnaire-8 (PHQ8) (Kroenke et al. 2009; Kroenke et al. 2010), Generalized Anxiety Disorder-7 (GAD-7) (Plummer et al. 2016), Minimal Documentation System (MIDOS2) (Stiel et al. 2010), Hornheider Screening Instrument (HSI) (Buchhold et al. 2020), and the Distress Thermometer (Ownby et al. 2019; Snowden et al. 2011). In addition, it also assesses the patient's subjective need for palliative or psycho-oncological support (for a detailed sample of all ePROM questions and possible answers, refer to our supplements). Specifically, patients could respond to the question, "Do you feel the need for psycho-oncological/palliative support?" with either "yes" or "yes, later." Both responses were considered positive. The **PHQ-8** assesses the severity of depressive symptoms using eight items rated on a four-point Likert scale (0 = not at all, 1 = some days, 2 = more than half the days, 3 = nearly every day) with a score range from 0 to 27. The **GAD-7** evaluates anxiety and fears experienced over the past two weeks through seven questions. Responses are rated on a four-point Likert scale (0 = not at all, 1 = at some days, 2 = more than half the days, 3 = nearly every day), with scores ranging from 0 to 21. Scores of 5–9 indicates mild anxiety, and scores > 15 indicate severe anxiety. The **MIDOS2**, a German version of the Edmonton Symptom Assessment Scale, includes ten questions assessing various symptoms on a four-point Likert scale (0 = no symptoms, 1 = mild symptoms, 2 = moderate symptoms, 3 = severe symptoms). Additionally, patients’ subjective general well-being is categorized from “very poor” (4) to “very good” (0), with the possibility to include additional symptoms. The **HSI** comprises seven questions assessing physical and mental health, including questions about disease and treatment awareness and family support. Responses to some items are binary (“yes” or “no”), while others are ranked numerically from “0” (rather good) to “2” (rather poor). When referring to “physical", "mental" or "general" condition, it pertains to the patient's subjective state over the past three days. A patient is considered in need of care if their score exceeds 4, with a maximum sum score of 14. The **Distress Thermometer** prompts patients to quantify their distress level over the past ten days on a scale from 0 (no distress) to 10 (very distressed lately). A cut-off value of ≥ 5 indicates clinically significant distress. For analysis purposes, "good" included "rather good" and "very good”, while the designation "poor" included "rather poor" and "very poor".

### Statistical Analysis

Data management and statistical analyses were conducted using SPSS Statistics version 29.0 (IBM, New York). We used descriptive analyses including medians, means, and standard deviations. Group differences were determined using analysis of variance (ANOVA), T-Tests for independent samples, and non-parametric tests such as the Chi-Square Test (χ^2^) and Mann–Whitney U Test. Furthermore, we used multiple logistic regression analyses to assess the relationship between symptom burden, as measured by ePROM responses, and the subjective need for palliative support. Correlation analyses were conducted using Pearson and Spearman methods. For all tests, a significance level of p < 0.05 was predetermined.

### Sum score and cut-off evaluation

A MIDOS2 sum score was created for the purpose of this study. For the cut-off analysis, we plotted the MIDOS2 sum score against both the subjective need for palliative support and survival status (less than or more than one year), using a receiver operating characteristic (ROC) curve (Zou et al. 2007). This allowed us to evaluate the MIDOS2 sum score’s predictive capability for patient survival and subjective palliative needs. A higher AUC indicates better discriminatory capability. Using the Youden Index, we calculated the optimal cut-off value, which identifies the threshold where both sensitivity and specificity reach their maximum (Ruopp et al. 2008). To potentially enhance sensitivity and specificity, symptoms significantly more intense in patients expressing a subjective need for palliative support were assigned double weight. A subsequent new ROC curve was created using this modified sum score, with cut-off values identified using the Youden-Index and sensitivity and specificity calculations.

## Results

A total of 265 patients were eligible for our study. Among these, 70 patients survived less than one year, while 195 patients survived beyond a year after screening (Fig. 1). Of these, 143 patients (54.0%) completed the ePOS questionnaire in its entirety, while 122 patients (46.0%) skipped at least one question.

### Patient characteristics and general symptom burden

The main characteristics of the 265 patients, along with their self-reported symptom burden, are summarized in Table 1. Only 17 patients (6.4%) received an inpatient SPC consultation. Among the reported symptoms, tiredness emerged as the most prevalent, with 37.9% of patients rating it as moderate or severe. This was followed by pain (27.6%), weakness (27.4%), anxiety (24.5%), and depressive symptoms (14.3%), all of which were frequently reported as moderate or severe.

**Table 1:** Patient characteristics according to the subjective need for support

		Total n = 265	SPC Yes n = 55	SPC No n = 181
Sex n (%)	Female Male	109 (41.1) 156 (58.9)	27 (49.1) 28 (50.9)	67 (37.0) 114 (63.0)
Age at diagnosis (SD)		53.92 (± 14.64)	51.53 (± 13.18)	53.65 (± 14.41)
Age at death (SD) **		58.72 (± 14.29)	55.92 (± 11.84)	58.76 (± 14.37)
Tumour entity n (%)	Nervous system Gastrointestinal Dermatological Urogenital Lung Sarcoma Breast Endocrinological Others	63 (23.8) 42 (15.8) 57 (21.5) 25 (9.4) 29 (10.9) 17 (6.4) 7 (2.6) 13 (4.9) 12 (4.7)	15 (27.3) 9 (16.4) 6 (10.9) 5 (9.1) 4 (7.3) 4 (7.3) 2 (3.6) 6 (10.9) 4 (7.2)	41 (22.7) 31 (17.1) 42 (23.2) 17 (9.4) 23 (12.7) 12 (6.6) 4 (2.2) 6 (3.3) 5 (2.8)
Metastases n (%)	Pulmonary Hepatic Bone Visceral Cerebral	56 (21.1) 51 (19.2) 57 (21.5) 28 (10.6) 65 (24.5)	16 (29.1) 12 (21.8) 15 (27.3) 7 (12.7) 16 (29.1)	34 (18.8) 35 (19.3) 37 (20.4) 19 (10.5) 38 (21.0)
SPC consultation n (%)		17 (6.4)	6 (10.9)	8 (4.4)
Physical condition n (%)	Very poor Rather poor Moderate Rather good Very good	0 (0.0) 37 (14.1) 127 (48.5) 97 (36.6) 1 (0.4)	0 (0.0) 14 (25.5) 30 (54.5) 11 (20.0) 0 (0.0)	0 (0.0) 19 (10.6) 83 (46.1) 77 (42.8) 1 (0.6)
Mental condition n (%)	Very poor Rather poor Moderate Rather good Very good	2 (0.8) 45 (17.1) 118 (44.9) 98 (37.3) 0 (0.0)	0 (0.0) 17 (30.9) 28 (50.9) 10 (18.2) 0 (0.0)	2 (1.1) 23 (12.7) 78 (43.1) 78 (43.1) 0 (0.0)
General well-being n (%)	Very poor Rather poor Moderate Rather good Very good	2 (0.8) 30 (11.7) 108 (42.0) 104 (40.5) 13 (5.1)	2 (3.6) 12 (21.8) 27 (49.1) 12 (21.8) 2 (3.6)	0 (0.0) 16 (8.9) 70 (38.9) 83 (46.1) 11 (6.1)

*At time of the study

**If deceased

### ROC curve, cut-off analysis using Youden Index, sensitivity and specificity

**Fig. 2:**
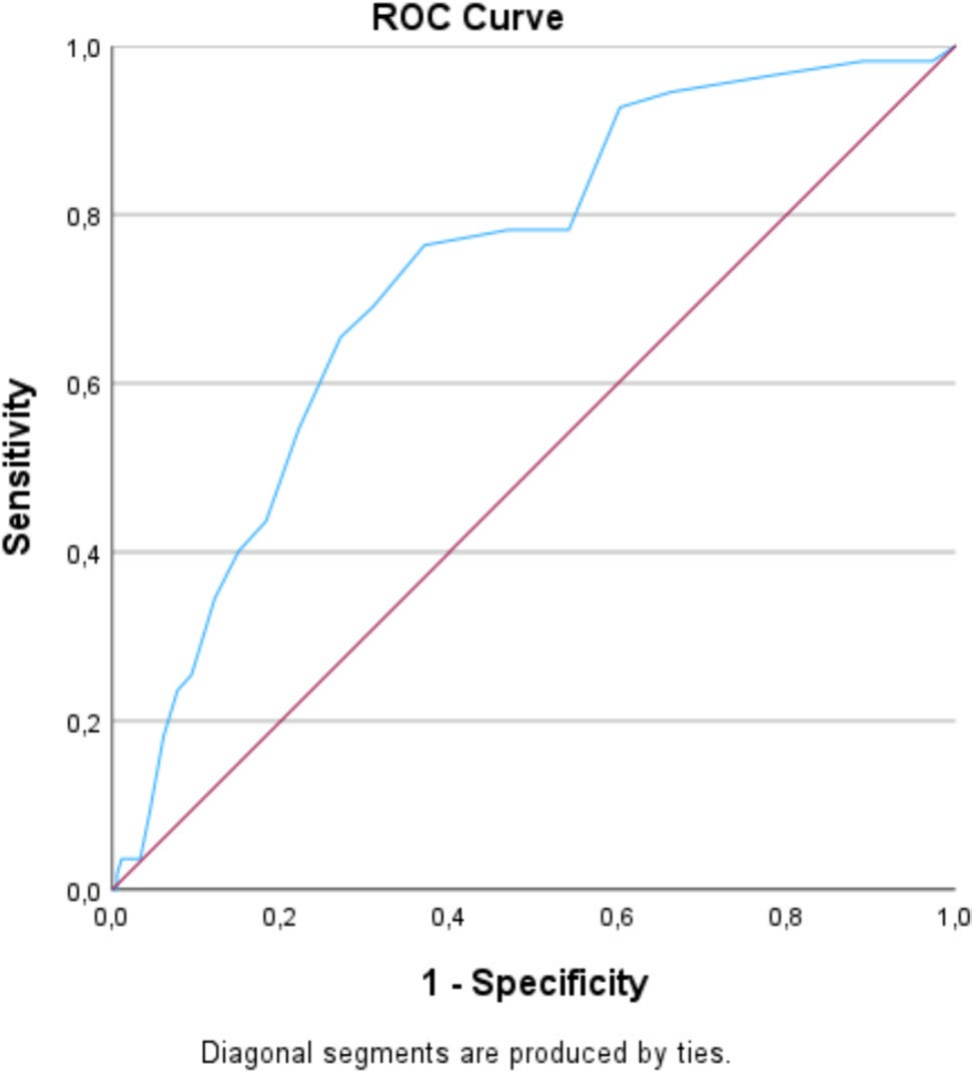
ROC curve for evaluating the MIDOS2 sum score in relation to the subjective need for palliative support

**Fig. 3:**
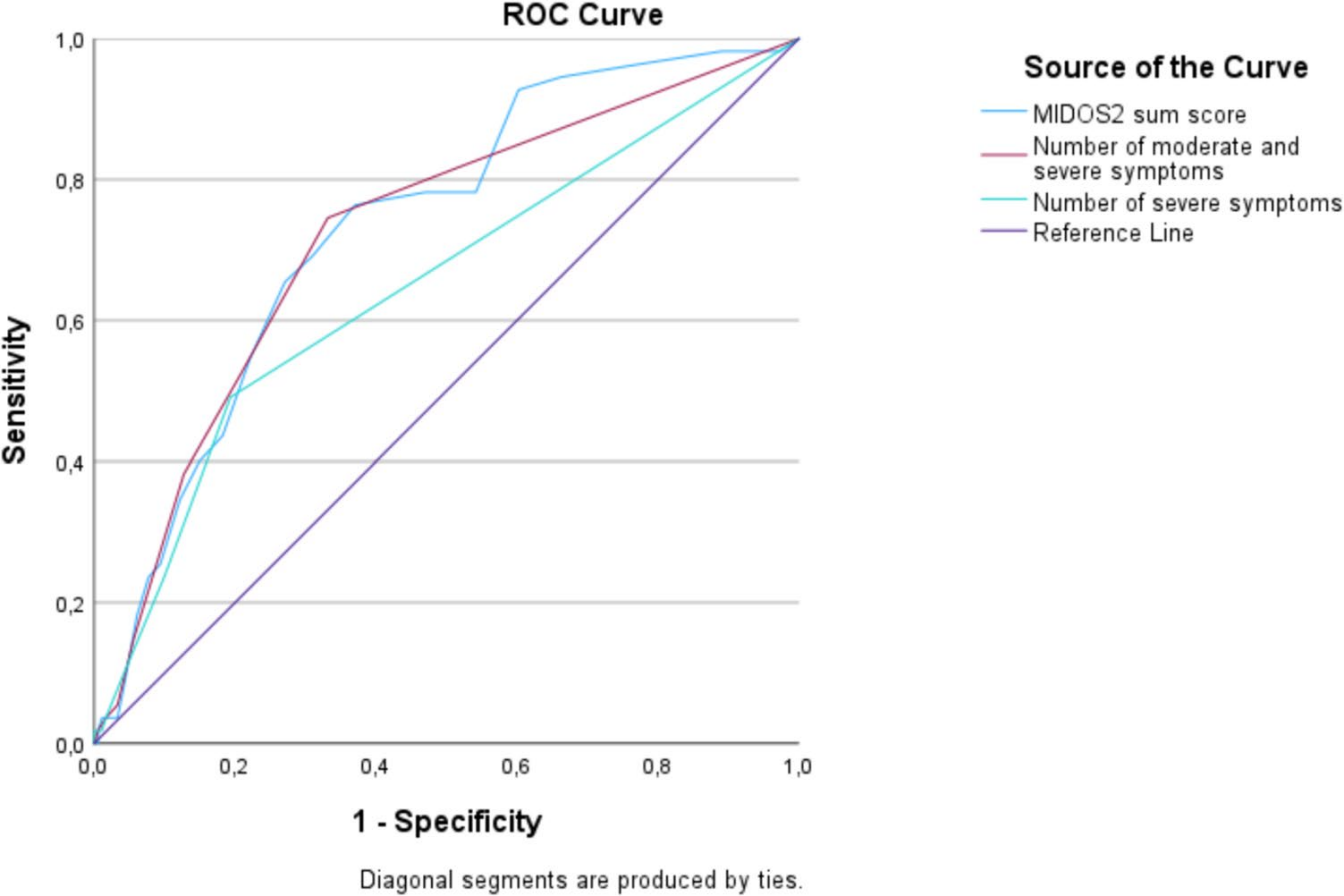
ROC curves for evaluating different approaches in relation to the subjective need for palliative support

The ROC curves illustrating the relationship between the true positive rate (sensitivity) and the false positive rate (1-specificity) at different threshold values when plotting the MIDOS2 sum score against the subjective need for palliative support (Fig. 2). Using this approach, we obtained an AUC of 0.732, with the highest Youden-Index at a threshold value of 8 points, resulting in a sensitivity of 76.36% and a specificity of 62.98%. Additionally, we conducted a comparative analysis using self-reported symptoms classified as severe ("number of severe symptoms") and the combination of moderate and severe symptoms ("number of moderate and severe symptoms") in the MIDOS2 instead of the MIDOS2 sum score. These approaches resulted in lower AUC values in each case (Fig. 3 and Table 2). Due to the lower AUC value, we did not calculate the cut-off value, sensitivity and specificity for this approach. Given that the symptoms pain, weakness, anxiety, tiredness, depressive symptoms, and general well-being were significantly more intense in patients expressing a subjective need for palliative support, we double weighted these symptoms. Thus, a cut-off value of 14 and higher sensitivities and specificities were achievable (Table 3).

**Table 2:** AUC of different approaches in relation to the subjective need for palliative support

Test result variable(s)	AUC
MIDOS2 sum score	0.732
Number of moderate and severe symptoms	0.721
Number of severe symptoms	0.647

**Table 3:** Results of the MIDOS2 sum score cut-off analysis for palliative support

	AUC*	Youden-Index	Cut-off	Sensitivity (%)	Specificity (%)
MIDOS2 sum score	0.732	0.393	8	76.36	62.98
MIDOS2 sum score weighted × 2 **	0.741	0.406	14	78.18	72.38

*Area under the ROC curve

**Double weighted significant Items (weakness, depressive symptoms, anxiety, tiredness, pain and general well-being)

**Fig. 4:**
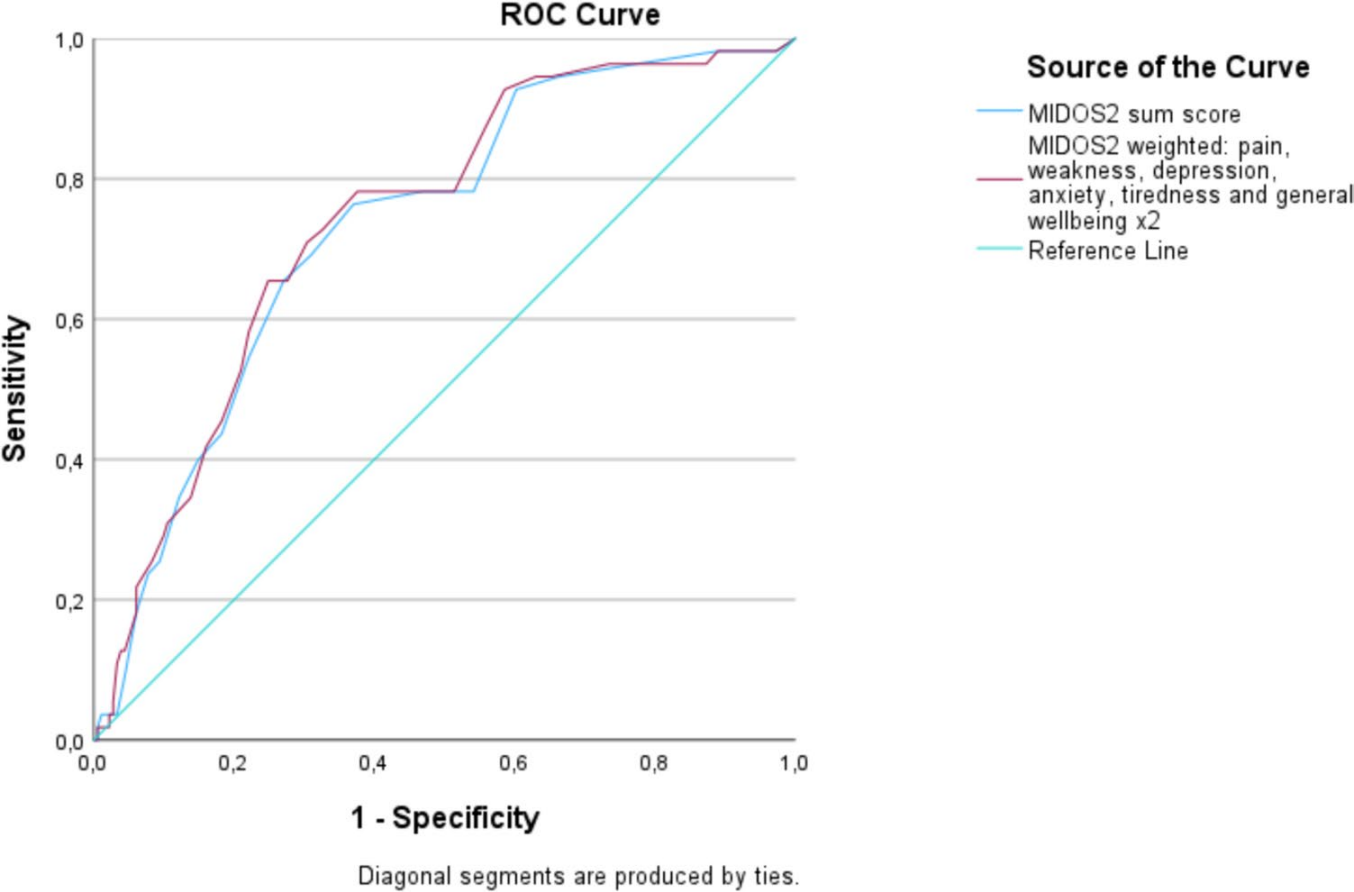
ROC curves of different approaches for a survival of less than one year after screening

Additionally, we also generated a ROC curve to assess the MIDOS2 sum score in relation to a survival of less than one year (Fig. 4). The AUC was 0.588. Due to this relatively modest value, we opted against further analysis of the cut-off value, sensitivity, and specificity of the MIDOS2 sum score concerning survival time.

### Patient’s need for specialised palliative care related to symptom burden

In total, 55 patients (20.8%) expressed a need for support from the palliative care team. There were no significant differences between patients who felt the need for palliative support and those who did not in terms of gender (χ^2^ = 2.566, p = 0.118) or tumour entity (χ^2^ = 18,858, p = 0.092).

**Fig. 5:**
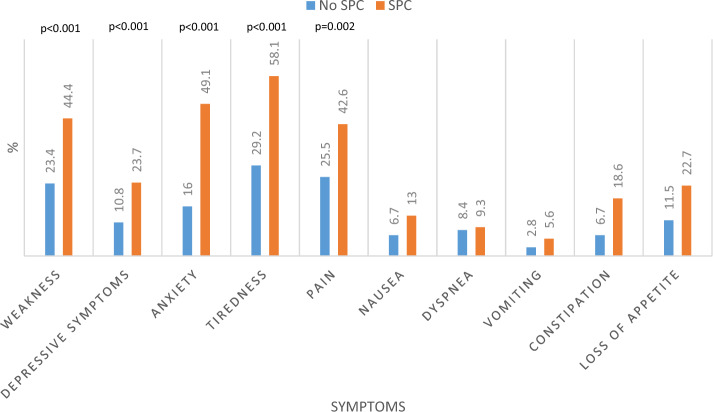
Symptoms reported as moderate/severe according to subjective need for palliative support using MIDOS2. Mann–Whitney U Test showed a significant difference in the intensity of the symptoms weakness (p < 0.001), depressive symptoms (p < 0.001), anxiety (p < 0.001), tiredness (p < 0.001) and pain (p = 0.002)

Comparatively, patients who felt the need for palliative support reported significantly higher intensities of symptom in weakness (p < 0.001, U = 3354.500, Z = − 3.600), depressive symptoms (p < 0.001, U = 3513.000, Z = − 3.538), anxiety (p < 0.001, U = 3001.500, Z = − 4.527), tiredness (p < 0.001, U = 3374.000, Z = − 3.664), and pain (p = 0.002, U = 3600.000, Z = − 3.071) compared to those without the subjective need for palliative support (Fig. 5).

**Fig. 6:**
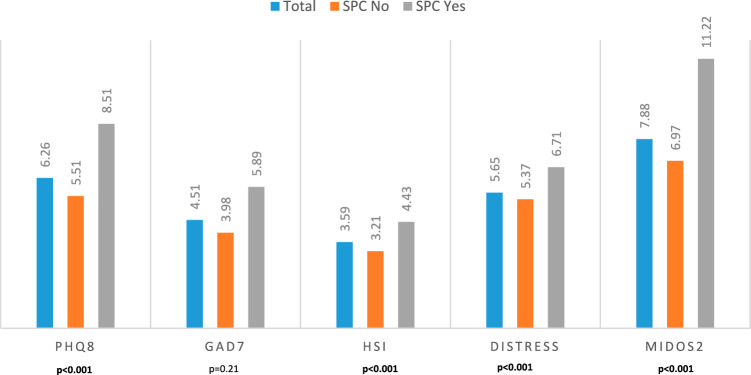
Mean of symptom scores according to the patient’s subjective need for support. ANOVA showing a significant difference in the sum scores of the questionnaires PHQ8 (p < 0.001), HSI (p < 0.001), Distress-Thermometer (p < 0.001) and MIDOS2 (p < 0.001)

The multiple logistic regression analysis showed that symptom intensity, specifically anxiety (B = − 0.534, Wald = 4.839, Exp (B) = 0.586, 95% CI = 0.364–0.943, p = 0.028) and pain (B = − 0.451, Wald = 4.094, Exp (B) = 0.637, 95% CI = 0.412–0.986, p = 0.043), positive predicted the decision to obtain palliative support. Furthermore, comparing questionnaire scores, ANOVA showed significant differences in the sum scores of the PHQ8 (p < 0.001), HSI (p < 0.001), Distress-Thermometer (p < 0.001), and MIDOS2 (p < 0.001) between patients who felt a need for palliative support and those who did not (Fig. 6).

There was no significant correlation between the physical (p = 0.397), mental (p = 0.155) or general (p = 0.977) condition and the performance Status (ECOG) in our patient’s population.

### Patient’s need for palliative support related to subjective physical, general, and mental health condition

**Fig. 7:**
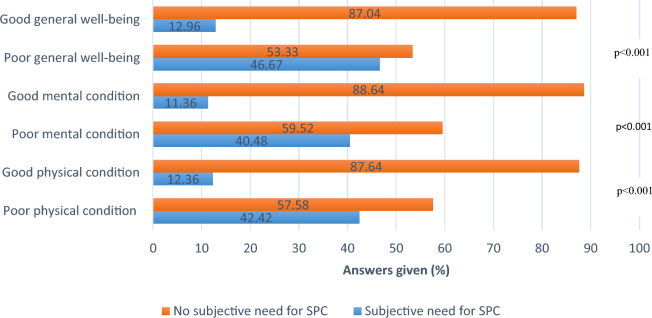
Differences in subjective need for palliative support according to the patient’s current condition. Significant differences on various levels of the patient´s health of those patients with the subjective need for palliative support and those without (p < 0.001)

A total of 40.0% (12 out of 30) of patients reporting a rather poor physical condition felt the need for palliative support, while 43.8% (14 out of 32) with very poor or rather poor general well-being expressed a desire for a consultation with the palliative care team. Significant differences were observed in self-reported well-being: both physical and mental conditions of patients expressing a subjective need for palliative support were reported as poorer compared to those who did not express such a need (p < 0.001). Additionally, the general well-being was subjectively lower in patient’s subjective need of palliative support (p < 0.001) (Fig. 7).

### Survival analysis: patient’s subjective need of palliative support and symptom burden

No significant differences were observed in terms of gender (χ^2^ = 0.792, p = 0,461) or tumor entity (χ^2^ = 17.954, p = 0.117) between patients with a survival of less than one year and those who survived more than a year. Similarly, most sum scores, including PHQ-8 (p = 0.85), GAD-7 (p = 0.28), HSI (p = 0.56), and Distress-Thermometer (p = 0.28) did not show significant differences between the two groups. However, there was a significant difference in symptom intensity assessed by MIDOS2 (p = 0.013). Specifically, the mean MIDOS2 sum score was higher (9.35 (SD ± 6.26)) among patients with a survival of less than one year and those who survived more than a year (7.34 (SD ± 5.21)). The Mann–Whitney U Test revealed significant differences in symptom intensity for constipation (U = 4075.500, Z = − 2.803, p = 0.005), weakness (U = 4145.500, Z = − 2.084, p = 0.037), loss of appetite (U = 2481.500, Z = − 2.274, p = 0.023), and general well-being (U = 4355.500, Z = − 2.057, p = 0.040). Notably, all these symptoms were rated higher in patients with a survival of less than one year. Lastly, patients who with a survival of less than one year exhibited a significantly higher need for palliative support compared to those who survived more than a year (p < 0.001).

## Discussion

The use of validated symptom assessment tools and PROMs to initiate SPC referrals based on patients’ needs remains an area of active research. SPC referrals based on patients’ needs are widely accepted, particularly due to the frequent occurrence of multiple uncontrolled physical symptoms and unmet psychosocial needs in ACP (Vanbutsele et al. 2018; Vogt et al.^2021^; Tewes et al. 2018a, b).

This study represents the first attempt to conduct a cut-off analysis using the MIDOS2 sum score to determine subjective patient needs for palliative support, demonstrating favourable sensitivity and specificity (Table 2). Incorporating a weighted score based on the prevalent self-reported symptoms pain, weakness, anxiety, tiredness, depressive symptoms, and general well-being potentially higher sensitivity and specificity were achievable. In the aforementioned German study (Braulke et al. 2023), IPOS and MIDOS2 were used as screening assessment tools, unveiling that nearly 60% of patients screened positive, indicative of unmet needs for SPC. The advantages of the MIDOS2 tool included the perception as a useful in sensitizing healthcare providers to early SPC integration and the consideration as a user-friendly, low-threshold screening instrument for assessing SPC necessity. Noteworthy, it seems that such a concise screening approach may prove sufficient compared to larger multidimensional screenings, which are often more time-consuming and challenging to implement daily (Solar et al. 2023).

The MIDOS2 sum score and cut-off used in this study focuses on the subjective need for palliative support, an area that remains relatively understudied. Research among oncology patients suggested that those experiencing high symptom burdens or psychological distress are more likely to perceive a need for palliative support. However, they may refrain from explicitly requesting it and instead follow their oncologist’s advice (Schenker et al. 2014). This observation is interesting, particularly given that most patients facing incurable diseases seems to wish to be informed from their healthcare provider regarding their diagnosis, prognosis, and available options concerning SPC (De Vleminck et al. 2015). Another study involving oncological inpatients outlined that most patients exhibited a moderate to weak level of understanding and harbored substantial misinformation about PC (Atena et al. 2021). This could influence patients’ answers when using ePROMs, as many patients may be unsure about the role and benefits of PC, and may hold erroneous or negative beliefs (Masel et al. 2016). The importance of face-to-face communication, alongside the implementation of skills and strategies to facilitate early referral to SPC, should not be underestimated (Collins et al. 2022). These findings underscore the need for further exploration into the preferences of ACP regarding palliative support, as well as the implications of offering SPC through ePROMs compared to personal conversation, which are beyond the scope of this study.

Our findings indicate that only 6.4% of patients received an SPC referral, despite enduring significant physical and mental burdens. Although 55 patients (20.8%) felt the need of palliative support, only six were referred, consistent with earlier research conducted by our team (Tewes et al. 2018a, b). This highlights an enormous disparity between external assessments and patients’ subjective needs for PC. It is noteworthy that the decision to involve the palliative care team was at the discretion of the treating healthcare providers.

To the best of our knowledge, our study is the first establishing an association between poor general and physical condition, as well as higher general symptom burden (assessed via PHQ-8, HSI, Distress Thermometer, and MIDOS2), and the inclination toward palliative support. Additionally, patients with a subjective need for palliative support exhibited significantly higher levels of weakness, depressive symptoms, anxiety, tiredness, and pain compared to patients without such a need. These findings are consistent with a prior study conducted at our German Comprehensive Cancer Centre, which identified depressive symptoms, anxiety/fear, and weakness as predictors of perceived SPC needs in an outpatient setting (Tewes et al. 2018a, b). Similarly, Morita et al. showed that psychological distress was a primary reason leading to SPC referrals (Morita et al. 2008). Interestingly, in a recent study, Vogt et al. showed that 41% of ACP experiencing moderate or severe psychological distress, and 16.6% with mild or no psychological distress, wished to receive professional PC support (Vogt et al. 2021). Echoing these findings, our study identified severe anxiety and pain as significant predictors for the need for palliative support. Furthermore, it emphasizes the substantially heightened psychological distress among patients who felt the need for palliative support, underscoring the pivotal role of psychological burden in the context of ACP.

There were no differences between patients who felt the need for palliative support and those who did not, in terms of gender and tumour entity. Interestingly, ACP with a survival of less than one year after screening exhibited significantly higher symptom intensities measured by MIDOS2 and demonstrated a pronounced need for palliative support compared to those with a survival over one year (p < 0.001). This novel finding warrants further investigation and supports the current PC approach, which emphasizes tailored, individual, patient-centered care based on individual complexity. Such complexity is often higher during moments of crisis or at the end of life, where patients might experience the highest symptom burden and distress. In such scenarios, SPC plays a crucial role in providing personalized care aligned with patient needs, regardless of diagnosis or prognosis (van Oorschot et al. 2022; Carrasco-Zafra et al. 2020). Further research is needed to explore the integration of PROM and other clinical parameters, including laboratory data, to identify patients who could benefit the most from SPC (Müller et al. 2023).

## Limitations

Our investigation has several limitations. First, our retrospective analysis was conducted at a single centre including inpatients diagnosed with advanced cancer. Therefore, the generalizability of our findings may differ when compared to outpatient settings or patients receiving palliative care for non-oncological diseases. Second, because of the study design, the final sample size was modest. Third, using ePROM may exclude patients unable to complete this self-questionnaire due to high symptom burden, language barrier (our study exclusively included German-speaking participants), somnolence, or severely poor general condition. Fourth, our study did not address important aspects of PC such as family burden or social and spiritual needs. Fifth, the MIDOS2 cut-off analysis is based solely on the patient’s perspective and subjective need for palliative support, without input from experts or objective criteria. Sixth, MIDOS2 does not provide diagnostic capabilities but aims to identify patients with unmet PC needs; thus, the decision for SPC referral must be evaluated by healthcare professionals, albeit such evaluation does not guarantee referral. Seventh, the time interval between admission and screening varied, with a mean duration of 31.94 days. Eight, the analysis of PROMs is challenging due to missing data, nevertheless we decided to include all questionnaires because this approach leads to more representative results and higher transparency. Future studies should address missing data and consider appropriate methods for handling them. These several limitations underscore the necessity for cautious interpretation of our findings.

## Conclusion

The optimal timing of SPC referral among ACP remains unclear, often occurring late and thereby reducing the full benefits of PC. We stablished a cut-off value of eight points for the user-friendly screening tool MIDOS2. Particularly, the double-weighted MIDOS2 sum score showed high sensitivity and specificity, potentially aiding in the identification of patients with a subjective need for palliative support. Our study revealed a correlation between patient’s need for palliative support, higher physical burden, and increased psychological distress. Importantly, this subjective need for palliative support is independent of gender and tumor entity and increases in patients with a survival of less than one year. Further research in symptom screening and referral criteria is imperative to refine our understanding and optimize the delivery of PC.

### Supplementary Information

Below is the link to the electronic supplementary material.

Supplementary file1 (DOCX 22 kb)

## Data Availability

The authors confirm that the data supporting the findings of this study are available within the article and/or its related files. Any other information that support the findings of this study are available from the corresponding author, upon reasonable request.
