# Supplementary material for: Determining the cut-off value for the Minimal Documentation System (MIDOS2) screening tool to initiate specialized palliative care based on patient’s subjective need for palliative support and symptom burden in inpatients with advanced cancer
Source: J Cancer Res Clin Oncol. 2024 Jul 24;150(7):360. doi: 10.1007/s00432-024-05897-x (PMC11269497; doi:10.1007/s00432-024-05897-x)
Supplement: Supplementary file 1 — Supplementary file1 (DOCX 22 kb) [file 432_2024_5897_MOESM1_ESM.docx]

| **ePROM Questions** | **Possible answers** | | | | | | |
| --- | --- | --- | --- | --- | --- | --- | --- |
| What is your current family status? | Single | With partner, unmarried | Married | Living separately | Divorced | Widowed | Other |
| Do you have kids? | Yes | No |  |  |  |  |  |
| What is your highest school diploma? | Not in school yet | Elementary school | Graduation from polytechnic high school | Secondary school diploma | High school diploma | University degree | Other |
| Are you currently employed? | Yes | No |  |  |  |  |  |
| Are you currently taking tranquilizers, antidepressants or sleeping pills? | No | Yes, daily | Yes, sometimes |  |  |  |  |
| Have you been or are you currently undergoing psychological, psychiatric or psycho-therapeutic treatment? | Never before | Earlier, it lasted until (blank space to be filled out by the patient) | Currently under treatment |  |  |  |  |
| How did you feel physically during the past 3 days? | Rather good | Moderate | Rather bad |  |  |  |  |
| How did you feel mentally during the past 3 days? | Rather good | Moderate | Rather bad |  |  |  |  |
| Is there anything that is bothering you regardless of the current illness? | Yes | No |  |  |  |  |  |
| Do you have someone with whom you can talk about your worries and fears? | Yes | No |  |  |  |  |  |
| Is someone in your family particularly burdened by the hospital stay? | Yes | No |  |  |  |  |  |
| Can you find peace inside during the day? | Yes | No |  |  |  |  |  |
| How well do you feel informed about disease and treatment? | Rather good | Moderate | Rather bad |  |  |  |  |
| Please select a number on the thermometer (0-10) that best describes how stressed you have felt in the last week including today | Choice between 0 and 10 |  |  |  |  |  |  |
| Intensity of pain during the past week | No symptoms | Mild symptoms | Moderate  symptoms | Severe  symptoms |  |  |  |
| Intensity of nausea during the past week | No symptoms | Mild symptoms | Moderate  symptoms | Severe  symptoms |  |  |  |
| Intensity of vomiting during the past week | No symptoms | Mild symptoms | Moderate  symptoms | Severe  symptoms |  |  |  |
| Intensity of shortness of breath during the past week | No symptoms | Mild symptoms | Moderate symptoms | Severe  symptoms |  |  |  |
| Intensity of constipation during the past week | No symptoms | Mild symptoms | Moderate symptoms | Severe  symptoms |  |  |  |
| Intensity of weakness during the past week | No symptoms | Mild symptoms | Moderate symptoms | Severe  symptoms |  |  |  |
| Intensity of loss of appetite during the past week | No symptoms | Mild symptoms | Moderate symptoms | Severe  symptoms |  |  |  |
| Intensity of tiredness during the past week | No symptoms | Mild symptoms | Moderate symptoms | Severe  symptoms |  |  |  |
| Intensity of depression during the past week | No symptoms | Mild symptoms | Moderate symptoms | Severe  symptoms |  |  |  |
| Intensity of anxiety during the past week | No symptoms | Mild symptoms | Moderate symptoms | Severe  symptoms |  |  |  |
| Intensity of other feelings during the past week | No symptoms | Mild symptoms | Moderate symptoms | Severe  symptoms |  |  |  |
| What is your general wellbeing today? | Very poor | Poor | Moderate | Good | Very good |  |  |
| Were you restricted in your work or other day-to-day activities during the past week? | Not at all | Slightly | Moderate | A lot |  |  |  |
| Were you restricted in your hobbies or other leisure activities during the past week? | Not at all | Slightly | Moderate | A lot |  |  |  |
| Did you feel tense during the past week? | Not at all | Slightly | Moderate | A lot |  |  |  |
| Have you been worried during the past week? | Not at all | Slightly | Moderate | A lot |  |  |  |
| Have you been irritable during the past week? | Not at all | Slightly | Moderate | A lot |  |  |  |
| Did you feel depressed during the past week? | Not at all | Slightly | Moderate | A lot |  |  |  |
| During the past 2 weeks, how often did you feel little interest or pleasure in what you were doing? | Not at all | Some days | More than half of the days | Almost every day |  |  |  |
| During the past 2 weeks, how often did you feel depressed, melancholy, or hopeless? | Not at all | Some days | More than half of the days | Almost every day |  |  |  |
| During the past 2 weeks, how often did you have difficulty falling asleep, staying asleep, or sleeping more? | Not at all | Some days | More than half of the days | Almost every day |  |  |  |
| During the past 2 weeks, how often did you feel fatigue or a lack of energy? | Not at all | Some days | More than half of the days | Almost every day |  |  |  |
| During the past 2 weeks, how often did you feel a decrease in appetite or an excessive need to eat? | Not at all | Some days | More than half of the days | Almost every day |  |  |  |
| During the past 2 weeks, how often have you felt affected by a low opinion of yourself, a sense of failure, or disappointment in your family? | Not at all | Some days | More than half of the days | Almost every day |  |  |  |
| During the past 2 weeks, how often did you feel impaired by difficulty concentrating on something, for example, reading the newspaper or watching television? | Not at all | Some days | More than half of the days | Almost every day |  |  |  |
| During the past 2 weeks, how often did you feel affected by the slowing down of your speech or movements or by a strong urge to move and restlessness? | Not at all | Some days | More than half of the days | Almost every day |  |  |  |
| During the past 2 weeks, how often did you feel affected by thoughts that you would rather be dead or that you wanted to harm yourself? | Not at all | Some days | More than half of the days | Almost every day |  |  |  |
| During the past 2 weeks, how often did you feel nervous, anxious or tense? | Not at all | Some days | More than half of the days | Almost every day |  |  |  |
| During the past 2 weeks, how often did you feel affected by the feeling of not being able to stop or control your worries? | Not at all | Some days | More than half of the days | Almost every day |  |  |  |
| During the past 2 weeks, how often did you feel affected by the feeling of excessive worries concerning various matters? | Not at all | Some days | More than half of the days | Almost every day |  |  |  |
| During the past 2 weeks, how often did you feel affected by difficulties in relaxing? | Not at all | Some days | More than half of the days | Almost every day |  |  |  |
| During the past 2 weeks, how often did you feel affected by restlessness, making it difficult to sit? | Not at all | Some days | More than half of the days | Almost every day |  |  |  |
| During the past 2 weeks, how often did you feel quickly annoyed or irritated? | Not at all | Some days | More than half of the days | Almost every day |  |  |  |
| During the past 2 weeks, how often did you feel affected by the feeling of fear, like something bad was going to happen? | Not at all | Some days | More than half of the days | Almost every day |  |  |  |
| Do you feel the need for psycho-oncological support? | No | Yes, later | Yes |  |  |  |  |
| Do you feel the need for palliative support? | No | Yes, later | Yes |  |  |  |  |
| On which topics / occasions may we contact you by email? | New surveys in the course | Offers for people with cancer |  |  |  |  |  |

**Supplement.** Detailed sample of all ePOS (electronic psycho-oncological and palliative care screening) questions and possible answers.
